# A Vest-Pocket Medical Lexicon: Being a Dictionary of the Words, Terms, and Symbols of Medical Science

**Published:** 1865-05

**Authors:** 


A Vest-Pocket Medical Lexicon: Being a Dictionary of the Words, Terms, and Symbols of Medical Science. Collated from the best Authorities, with the addition of New Words, not before introduced into a Lexicon. With an Appendix. By D. B. St. John Rosa, M.D., Aural Surgeon to the New York Eye and Ear Infirmary. New York: Wm. Wood & Co., 61 Walker Street. 1865.

This is truly a pocket lexicon, being only about two inches square and three-quarters of an inch thick, embracing 363 pages. It is just what its title indicates; and will be found exceedingly convenient, especially by students of medicine.

For sale by W. B. Keen & Co., Lake St., Chicago.

